# Tracking and visualization of the sensing area for a tethered laparoscopic gamma probe

**DOI:** 10.1007/s11548-020-02205-z

**Published:** 2020-06-16

**Authors:** Baoru Huang, Ya-Yen Tsai, João Cartucho, Kunal Vyas, David Tuch, Stamatia Giannarou, Daniel S. Elson

**Affiliations:** 1 The Hamlyn Centre for Robotic Surgery, Department of Surgery and Cancer, Imperial College London, London, SW7 2AZ UK; 2 Lightpoint Medical Ltd, Chesham, UK

**Keywords:** Image-guided surgery, Prostate cancer, Tethered laparoscopic gamma probe, Minimally invasive surgery, Pose estimation, Tracking

## Abstract

**Purpose:**

In surgical oncology, complete cancer resection and lymph node identification are challenging due to the lack of reliable intraoperative visualization. Recently, endoscopic radio-guided cancer resection has been introduced where a novel tethered laparoscopic gamma detector can be used to determine the location of tracer activity, which can complement preoperative nuclear imaging data and endoscopic imaging. However, these probes do not clearly indicate where on the tissue surface the activity originates, making localization of pathological sites difficult and increasing the mental workload of the surgeons. Therefore, a robust real-time gamma probe tracking system integrated with augmented reality is proposed.

**Methods:**

A dual-pattern marker has been attached to the gamma probe, which combines chessboard vertices and circular dots for higher detection accuracy. Both patterns are detected simultaneously based on blob detection and the pixel intensity-based vertices detector and used to estimate the pose of the probe. Temporal information is incorporated into the framework to reduce tracking failure. Furthermore, we utilized the 3D point cloud generated from structure from motion to find the intersection between the probe axis and the tissue surface. When presented as an augmented image, this can provide visual feedback to the surgeons.

**Results:**

The method has been validated with ground truth probe pose data generated using the OptiTrack system. When detecting the orientation of the pose using circular dots and chessboard dots alone, the mean error obtained is $0.05^{\circ }$ and $0.06^{\circ }$, respectively. As for the translation, the mean error for each pattern is 1.78 mm and 1.81 mm. The detection limits for pitch, roll and yaw are $360^{\circ }, 360^{\circ }$ and $8^{\circ }$–$82^{\circ }\cup 188^{\circ }$–$352^{\circ }$ .

**Conclusion:**

The performance evaluation results show that this dual-pattern marker can provide high detection rates, as well as more accurate pose estimation and a larger workspace than the previously proposed hybrid markers. The augmented reality will be used to provide visual feedback to the surgeons on the location of the affected lymph nodes or tumor.

## Introduction

**Fig. 1:**
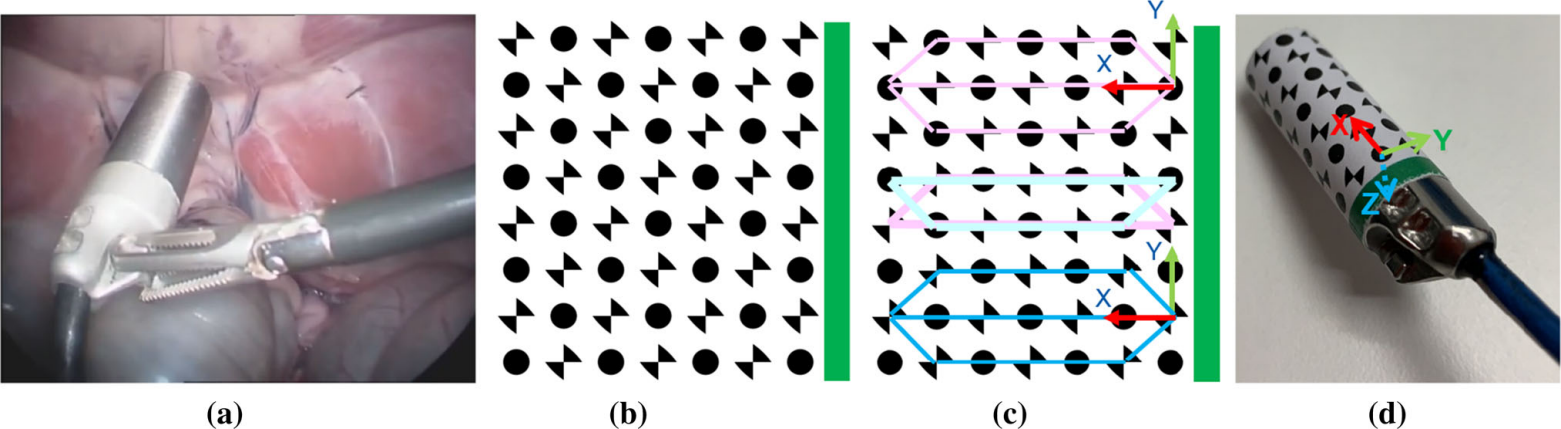
**a** An example of a tethered probe being used in MIS; **b** the gamma probe marker; **c** example detected circular dots and chessboard vertices; **d** the local coordinates defined on the probe

According to Cancer Research UK, prostate cancer is reported as one of the most common cancers in men in the UK with 47,700 new cases and 11,500 deaths reported each year [1]. One of the main treatment options for this cancer is surgery, and minimally invasive surgery (MIS) including robot-assisted procedures are increasingly used due to its significant advantages, such as reducing the risk of infection and trauma to the patient’s tissues [2]. Making a clear distinction between cancerous and non-cancerous tissue is an arduous task. Currently, surgeons still rely on their naked eye and sense of touch to detect where the cancer is located in the tissue. To address the compromised vision and tactile feedback in MIS, Lightpoint Medical Ltd. has developed a miniaturized cancer detection probe for MIS, called ‘SENSEI^®^’ (see Fig. 1a). This tethered laparoscopic probe relies on the cancer-targeting ability of established nuclear probes to identify the cancerous regions of the tissue more accurately [3].

The use of such a probe presents a visualization challenge, since the probe may not be in contact with tissue during the surgery, which makes it difficult to detect the location of the sensing area on the tissue surface. Additionally, when scanning a tissue, the surgeon needs to memorize the previously acquired probe data. This is inefficient, increases the surgeon’s workload and increases the probability of the cancerous tissue not being entirely removed or positive lymph nodes missed. Therefore, the development of a visualization tool that shows the surgeon directly where the cancerous tissue is located is of extreme importance.

To date, many probe tracking methodologies have been proposed. The first *in vivo* AR surgical anatomy visualization system with the probe tracked by an optical tracker was proposed in [4]. A magnetic tracking method was presented in [5] combined with stereoscopic video. However, the introduced additional tracking devices are likely to occupy valuable operating space and bring some intrinsic limitations such as line-of-sight and ferromagnetic interference. A commonly used approach is through laparoscopic image-based optical pattern detection which locates a pattern attached to a probe. Previous studies used corner detection to detect chessboard patterns attached to instruments [6, 7]. This method was extended in [8] by computing the probe pose with a randomly distributed fiducial pattern over the curved surface, which allowed the occlusion on fiducials and the outliers to be properly handled. Later, the circular dot pattern was proposed, which relied on a more efficient and robust ‘blob detector’ rather than the intersection of edges to estimate the pose of the instrument [9]. Zhang et al. [10] proposed a hybrid type, incorporating both aforementioned patterns, which provided more information when the ambiguous pose problems occurred. However, for the ‘SENSEI^®^’ used in this project, the rotation around its own axis does not affect the detection results since the probe is non-imaging. Therefore, these chessboard vertices are redundant.

In this paper, a new dual-pattern cylindrical marker is proposed to facilitate gamma probe tracking. The dual-pattern marker consists of circular dots and chessboard vertices which are simultaneously detected and tracked. To improve the robustness of the whole system and reduce the detection failures, temporal information is employed to complement marker detection. Our new marker and tracking framework are assessed using an OptiTrack system from where we collected the ground truth data. The detection rates, pose estimation accuracies and workspace coverage were calculated and we observed that using our novel dual-pattern marker we outperform the current state-of-the-art. The tissue surface is reconstructed using a structure from motion (SFM) algorithm and the intersection point between the surface and the probe axis is estimated. Using that intersection point, our framework highlights to the surgeon the part of the tissue that is being scanned.

## Methodology

### Dual-pattern marker design

In this paper, we proposed a dual-pattern marker (Fig. 1b) that combines the chessboard vertices and circular dots to estimate the instrument pose. The two patterns were equally spaced and placed circumferentially and appeared alternately. Every two lines of the pattern formed a trapezoidal shape and was considered as a detection unit (Fig. 1c) for pose estimation and tracking. A green stripe was placed at one end of the marker to resolve ambiguous pose and introduce asymmetry. The marker was attached to the cylindrical instrument such that the overall width matched the circumference, and the patterns were aligned with its axis.

A local coordinate frame was set at the surface of the probe (Fig. 1d), and its origin was regarded as the coordinate pivot. When the marker is flattened, the relative position of each feature in the X-Y coordinate frame can be determined from their size and separation. Thus, for a given radius of the probe, the 3D position ($P = [X, Y, Z]^\mathrm{T}$) of each dot and vertex in the 3D local coordinate frame can be determined from their 2D positions ($p = [x, y]^\mathrm{T}$).

### Feature detection

**Fig. 2:**
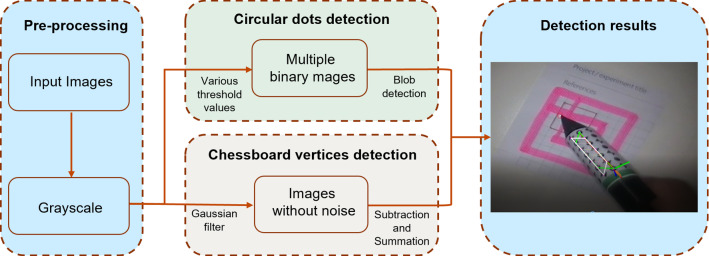
Feature detection algorithm workflow

The detection process of the proposed marker consists of two parts: blob detection and chessboard vertices detection. The detection algorithm workflow is shown in Fig. 2. For blob detection, a relatively simple algorithm for extracting circular blobs from images was used, called ‘SimpleBlobDectector’ in OpenCV. For the chessboard vertices detection (Fig. 2), a Gaussian filter was first applied to the grayscale image to eliminate noise and speckles, and then a robust and efficient detector called ‘Chess-board Extraction by Subtraction and Summation’ (ChESS) [11] was applied. To further filter spurious features that give weaker responses, an efficient non-maximum suppression method [12] was adopted to retrieve features with the maximum local responses. In addition, the area formed by the intersection of two lines at the center of the chessboard vertex was relatively easy to be misdetected as a dot. Hence, accurate detection of chessboard vertices would also help to eliminate incorrectly detected circular dots.

### Marker identification

The correspondences between the identified markers in the image and model points are necessary to conduct marker pose estimation. First, circular dots and chessboard vertices patterns are clustered based on their vicinity into different feature groups. The group with the largest number of features is used to find the trapeziums for transformation. The four endpoints located at the corners that form two trapeziums are identified from both vertex and dot patterns in this group. The trapezoidal shapes must be convex hulls and lie on the two parallel edges. Once the four vertices were identified, the pattern was transformed into a pattern in the image with the help of the corresponding information. Then, by comparing the transformed pattern and the projected pattern, the identity of each dot and vertex in the projected pattern can be the determined as the nearest point to the transformed pattern [10].

The addition of the green stripe introduces asymmetry to the markers which helps to identify the orientation of the marker frame. It was placed at the near side of the probe. For each iteration, the RGB image was converted to HSV to separate color from intensity which made it more robust to changes in lighting.

### Marker tracking

Once all the features that correspond to the model points have been identified, the pose of the probe can be estimated directly by computing a homography. The homography—i.e., the transformation that relates the markers and camera—can be estimated through $P_\mathrm{m} = HP_\mathrm{r}$ where $P_\mathrm{r}$ denotes the locations of points on the pattern expressed in a coordinate reference frame and $P_\mathrm{m}$ denotes the locations of the projected points on the camera image plane. During surgery, marker occlusion and invisibility are inevitable due to causes such as strong light reflections and blood staining. If the detection component fails to detect the whole marker and extract its location, the tracking method is used to complement the detection. In this tracking method, the optical flow is computed by the pyramidal affine Lucas–Kanade feature tracking algorithm [13] and temporal information is taken into consideration. By using the optical flow, the current position of the remaining features could be found. Then, the position of missing features could also be derived from the correspondence in the reference coordinate frame with the help of a homography. This homography can be estimated with only four pairs of non-collinear feature points, which indicates that it is robust to occlusion.

### Pose estimation

Once the position of the model points in the local coordinate frame of the marker and the corresponding projections on the image are found, a framework called infinitesimal plane-based pose estimation (IPPE) is employed [14], which is much faster than the current methods based on PnP and is more accurate in most cases. It returns a number of solutions and the geometric relationships of these solutions are clear. Normally, the correct solution will lead to a smaller re-projection error representing the difference between the tracked results and projections. Hence, in each video frame, the re-projection errors from both circular dots and chessboard vertices are compared and the pose with the smallest error should always be chosen. In this case, two solutions can be derived from each pattern, creating four solutions. If all of them give similar errors close to zero, then there is ambiguity. This situation typically happens when the marker is placed too far from or too close to the camera and the projection of the pattern is close to affine. Some methods are proposed to solve this issue, for instance [10] applies points from a different plane to create a large reprojection error for the wrong solution. However, the gamma probe collects gamma data from its tip and the rotation around the probe axis will not influence the detection results of the probe. The affine problem can be ignored as long as the re-projection error is sufficiently small.

### Augmented reality

The probe signals when the targeted tissue is detected, but it lacks the functionality to provide important visual feedback to the surgeon about the locations. Given the transformation matrix between the laparoscope and the local coordinate frame defined on the probe, the equation of the probe axis can be obtained from the geometrical relationship between the axis and the coordinate pivot. If the equation of the tissue surface is known then the intersection location between the probe axis and the tissue surface can be estimated. To this end, we used a functioning ‘SENSEI’ probe and a prostate phantom with a sealed radioactive Cobalt-57 source hidden inside. The diameter of the Cobalt-57 disk was 25 mm, and it was placed about 5 mm below the tissue surface. The experimental setup is shown in Fig. 6a, c. The ‘SENSEI’ probe was grasped with a laparoscope surgical grasper and the control unit nearby indicated the gamma counts. The laparoscope captured the video of the whole procedure with the image displayed on a monitor. The 3D reconstruction of the prostate phantom surface was conducted using SFM in MATLAB, and a corresponding surface point cloud was generated. The actual scale of this point cloud was calculated with the help of the ‘SENSEI’ probe of the known physical size. By calculating the distance between the points in the point cloud to the probe axis, points with short distances were determined. As the 3D reconstruction by SFM was quite dense, these points were considered to be the potential intersection points. Besides, the distance between the intersection point and the marker pivot point should be longer than the distance between the probe tip and the marker pivot.

### Experiments

#### Hardware setup

**Fig. 3:**
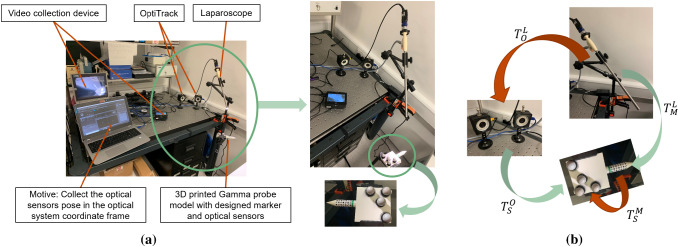
**a** Hardware setup for experiments; **b** the transformation matrixes between laparoscope, OptiTrack system, optical sensors and designed marker

Figure 3a shows the experimental setup illustrating a 3D printed model with the same dimensions as the real probe. During the detection procedure, the tip of the probe was positioned 2 to 3 cm from the tissue surface. Therefore, a cone with a height of 2 cm was added to the front end of the probe model to maintain a fixed distance to the tissue surface for validation. The designed marker was attached to the cylindrical probe, and four optical sensors were mounted on a flat plate attached to the model for validation via OptiTrack (NaturalPoint Inc, America). The diameter of the probe was 12 mm, and it can be placed directly into the patient’s abdominal cavity through standard MIS trocars. In this experiment, the probe could be placed in the view field of a standard 10 mm diameter monocular calibrated [15] laparoscope (KARL STORZ SE & Co. KG, Tuttlingen, Germany). The videos were displayed on a monitor and captured using a Ninja-2 box (Atomos Global Pty Ltd, Australia). The videos were streamed to a computer (2.5 GHz CPU, 8GB RAM) using S-Video to HDMI and HDMI to USB video converters (StarTech.com Ltd, America).

#### Pose estimation error

In order to validate the pose estimation algorithm, the OptiTrack system and its software, Motive, were used to obtain the ground truth and calculate the transformation matrix between the OptiTrack system and the optical sensors $T_\mathrm{S}^\mathrm{O}$. In addition, the marker pose in the laparoscope coordinate frame $T_\mathrm{M}^\mathrm{L}$ can be estimated; however, there were still two unknown registrations: the laparoscope to the OptiTrack system $T_\mathrm{O}^\mathrm{L}$ and optical sensors to the designed marker $T_\mathrm{S}^\mathrm{M}$. As shown in Fig. 3b, the green arrows indicate parameters that can be directly obtained while the red arrows represent the unknowns. The relationship between these four transformation matrixes is given as follows:

2.1 $$\begin{aligned} T_\mathrm{M}^\mathrm{L} \cdot T_\mathrm{S}^\mathrm{M} = T_\mathrm{O}^\mathrm{L} \cdot T_\mathrm{S}^\mathrm{O} \end{aligned}$$

This problem can be treated as an $AX = YB$ problem and 10 pairs of $T_\mathrm{M}^\mathrm{L}$ and $T_\mathrm{S}^\mathrm{O}$ were required to obtain the $T_\mathrm{S}^\mathrm{M}$ and $T_\mathrm{O}^\mathrm{L}$ [16]. However, the error from the registration accumulates in the final pose estimation error. During experimental validation, the probe was placed at the ‘typical’ position at 100 mm from the laparoscope to match a typical surgery. As there were two different patterns that could be detected on the marker, the final transformation matrix used was the one which led to a smaller re-projection error. For each pattern, 60 video trials were made and 10 of these were for registration to calculate $T_\mathrm{S}^\mathrm{M}$ and $T_\mathrm{O}^\mathrm{L}$ while 50 of these were for pose estimation error calculation. The position of the laparoscope and of the two OptiTrack cameras were always fixed. In every video trial, the probe was static, but the background of the scene was not static and changed over time. Besides, from trial to trial, the position of the probe was changed. In each trial, the relative pose between the ground truth and the estimated result was calculated as:

2.2 $$\begin{aligned} \mathrm{Relative\,pose\,matrix}= (T_\mathrm{S}^\mathrm{M})^{-1} \cdot (T_\mathrm{M}^\mathrm{L})^{-1} \cdot T_\mathrm{O}^\mathrm{L} \cdot T_\mathrm{S}^\mathrm{O} \end{aligned}$$

Ideally, the relative pose matrix should be equal to the identity matrix. However, this was not the case due to the error from the registration and pose estimation. The translation error was set as the mean of the fourth column in the matrix. To have a more intuitive understanding of the rotation error, the rotation matrix was converted to an axis-angle.

**Fig. 4:**
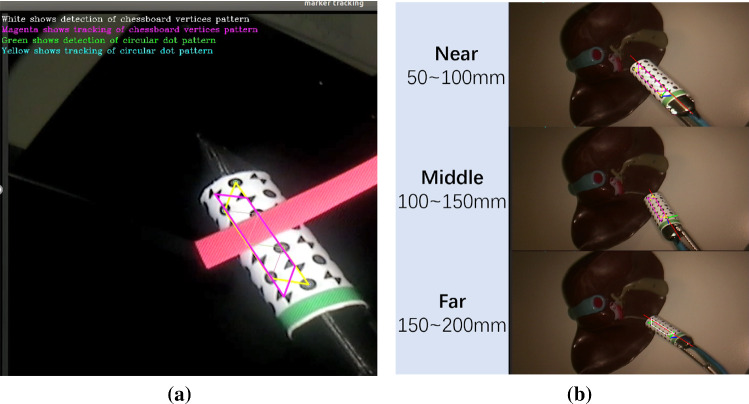
**a** Tracking results in the case of occlusion; **b** the experimental results for different testing distances between the probe and camera

**Table 1:** Summary of pose estimation error

Different marker	Translation mean error ± STD (mm)		Rotation mean error ± STD ($^{\circ }$)	
Our hybrid marker	Circular dots	Chessboard vertices	Circular dots	Chessboard vertices
	$1.78\pm 0.81$	$1.81\pm 0.80$	$0.05\pm 0.02$	$0.06\pm 0.02$
Previous hybrid marker [10]	$2.53\pm 1.40$		$0.69\pm 0.33$

**Table 2:** 3D tip distance when the cone tip is fixed

3D projection error			
Different marker	Mean error ± STD (mm)	Maximum error (mm)	Minimum error (mm)
Previous hybrid marker [10] with the failed frames	$17.17\pm 16.33$	137.72	0.00
Previous hybrid marker [10] without the failed frames	$1.73\pm 1.19$	5.41	0.00
Our hybrid marker	$0.22\pm 0.19$	1.90	0.00

**Table 3:** Maximum detectable distance and rotation angle around different axes

Rotation axis	Previous work [10]	Dual-pattern marker (ours)
Roll ($^\circ$)	$\pm \,85^\circ$	$360^ \circ$
Pitch ($^\circ$)	$\pm \,78^\circ$	$360^\circ$
Yaw ($^\circ$)	$\pm \,83^\circ$	$8^{\circ }$–$82^{\circ }\cup 188^{\circ }$–$352^{\circ }$
Distance to camera (mm)	60–200	50–220

**Fig. 5:**
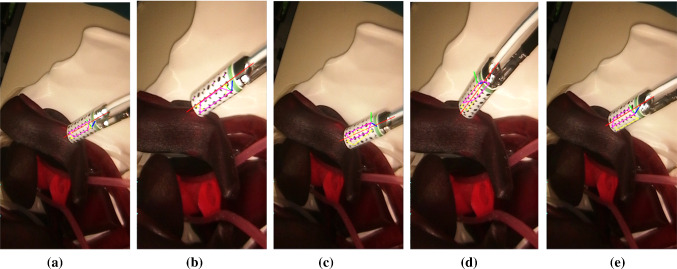
Examples where the pose estimation is more accurate by using **a** the circular dots pattern and **b** the chessboard vertices. Example where tracking failed for **c** the circular dots pattern and **d** the chessboard vertices. In **e** both vertices and dots pattern are detected in adjacent three marker lines

**Fig. 6:**
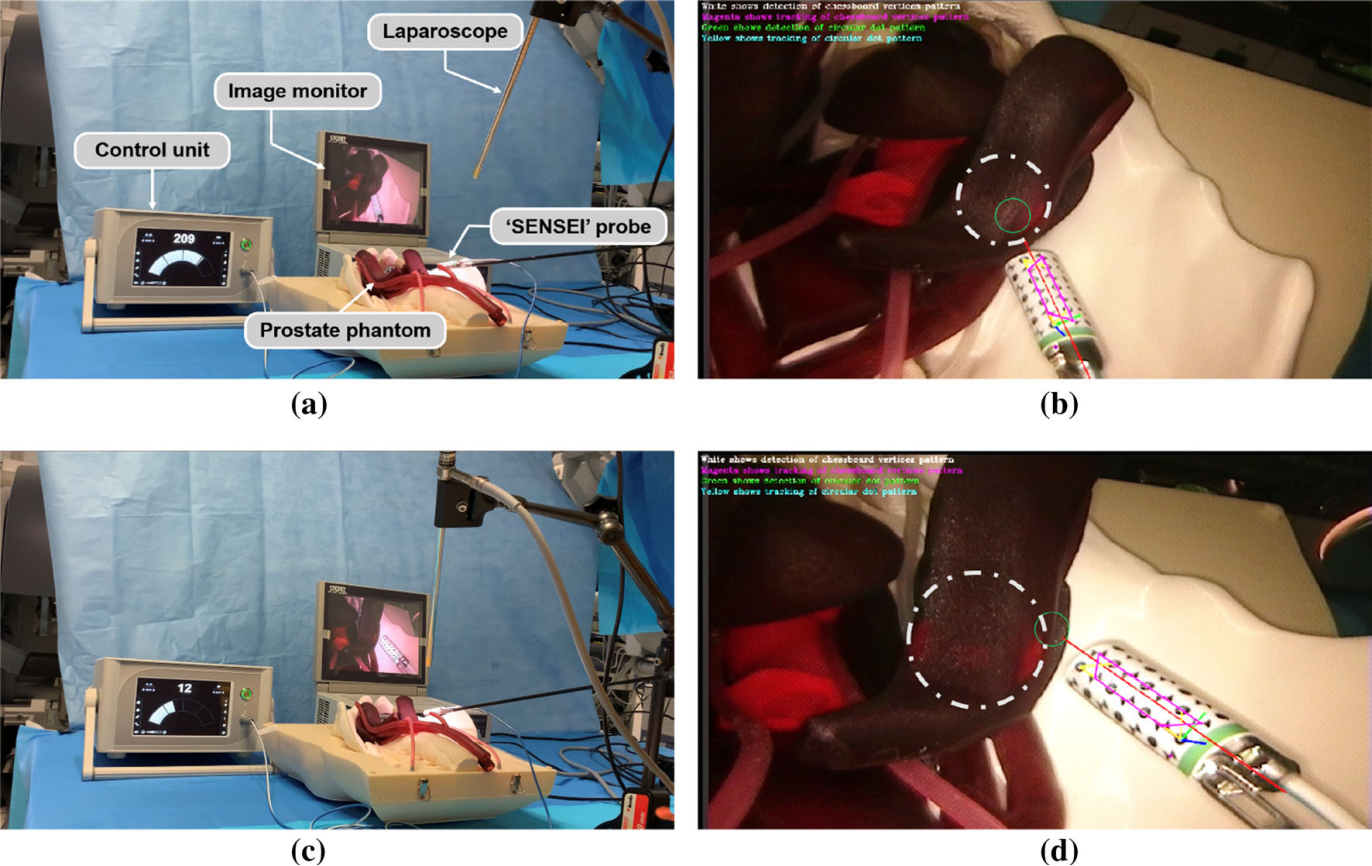
The hardware setup including laparoscope, image monitor, prostate phantom, ‘SENSEI’ probe, and control unit showing **a**, **b** a higher radiation level when the probe was pointing to and placed closer to the radioactive source; and **c**, **d** a lower radiation level when the probe was pointing to the edge of the source. The grey dashed circles in **b**, **d** show the position of radioactive Cobalt-57 source while the green circles represent the intersection area of the gamma probe axis and the tissue

#### Projection error

Given the geometric parameters of the probe and the transformation matrix from the camera to the marker, the 3D position of the cone tip simulating a 2 cm working distance could be estimated. The probe was rotated with a fixed tip position. However, because of the pose estimation error, the calculated 3D tip position was found to vary from frame to frame, with the distance between the tips in every two frames calculated as the projection error. The results were compared to previous hybrid marker [10], although in this case it could not be tracked during probe axial rotation around its own axis, resulting in large errors. Hence, the projection errors presented below for [10] were recorded with and without the failed frames.

#### Detection limit and detection rate analysis

For further validation, the detection limits and detection rates were calculated by recording the maximal experimentally detectable distance and rotation angle of the probe. The distance was recorded from the camera to the probe, and the limits of rotation were defined about the probe local coordinate axes (roll, pitch and yaw). When testing the distance limits, the probe was translated along the axis of the laparoscope until detection failed. To identify the rotational motion limits, the probe was placed 100 mm from the laparoscope, a typical distance for practical tissue scanning.

Since the detection of chessboard vertices relies on the intersection of edges, it was affected by image degrading effects like smudging and blooming. However, the circular dots detection algorithm was more robust because it did not rely on well-defined edge crossings. Regarding the dual-pattern marker detection, a frame was considered to be a success if either the chessboard vertices or circular dots pattern was detected, because they were independent of each other. In the experiments, the focus was set at the phantom surface and the probe was placed at different distances to the camera Fig. 4b: near (50–100 mm), middle (100–150 mm), far (150–200 mm).

## Experimental results and discussion

### Pose estimation error

Table 1 shows the validation results obtained from the dual pattern marker, which have a smaller mean error and a lower standard deviation than with the previous pattern. In addition, the pose estimation errors from the circular dots and the chessboard vertices patterns were quite similar and less than 2 mm, which means that both patterns worked well. Given the position of the model points defined in the local coordinate frame on the marker and the correspondence-tracked projections on the image, the pose of the marker was estimated by using the IPPE method. Specifically, the IPPE will give two affine poses for each pattern and will compare the results to select the one with the smallest reprojection error as the first output. This is why the newly designed pattern and new pose estimation algorithm can lead to the smaller mean error and increase the tracking accuracy.

### Projection error

It can be seen from Table 2 that for [10], the failure frames cause large projection errors unless the motion remained delicate. The errors calculated from our marker are lower due to pose estimation for every frame using two patterns.

### Detection and tracking analysis

The results of the detectable distance limits are shown in Table 3. The farthest distance at which the probe could be detected was 220 mm, and the marker works well between 50 and 150 mm, which is a reasonable working range for MIS. The maximum detectable angles are displayed in Table 3. Since the marker covered the entire probe surface circumferentially, detection results of the rotation around the roll axis are greatly improved. As the features in the marker are dense, the results when rotating around the pitch axis are also improved. As shown in Table 3, rotation around both roll and pitch axes can reach $360^\circ$. It is worth noting that the detectable angle range around the yaw axis is not $360^\circ$ since the axis of the probe was aligned with the axis of the laparoscope and the marker becomes invisible due to occlusion. Hence, there will be an angular range of about $16^\circ$ within which it is undetectable.

The detection rates for the near and middle distance ranges were 100%, which reduced to 99.7% when the probe was in the long distance range.

Since the pose estimations from chessboard vertices and circular dots are independent, if both of them are detected, the one with the smallest reprojection error will be selected. If identification of either fails, the system will rely on the other to get the probe pose. We list several different tracking scenarios in Fig. 5. Figure 5a shows a case where the pose estimation result from the circular dots pattern is more accurate than that from chessboard vertices, while Fig. 5b shows the opposite. In Fig. 5c, the circular dots pattern tracking failed so the probe pose is estimated from the vertices, while the opposite situation is presented in Fig. 5d. In Fig. 5e, both vertices and dots patterns are detected for three adjacent marker lines with the vertices pattern providing a more accurate pose estimation result.

### Tracking results for simulated occlusions

Figure 4a shows an example of an occlusion using a red stripe to block the markers. Although the number of remaining features was not enough to directly estimate the pose of the probe, they could still be used to calculate the homography. The position of the points that were occluded could then be inferred from the correspondence information between the coordinate reference frame and current camera image frame with the help of the homography. Therefore, the marker tracking enhanced the robustness of the entire system to occlusions.

### Augmented reality

Given a 3D point cloud representing tissue surface and the equation of the probe axis, the intersection point was estimated and the results are shown in Fig. 6. The red line indicates the axis of the probe, the grey dashed circle shows the position of radioactive Cobalt-57 source, and the green circle represents the intersection area of the gamma probe axis and the tissue. In Fig. 6a, b, the ‘SENSEI’ probe was close to and pointing towards the radioactive source, the probe recorded stronger gamma radiation of 209 counts per second. Figure 6c, d shows the opposite where the ‘SENSEI’ probe was pointing at the edge of the buried source, and the radiation was weak (12 counts per second). The AR system can therefore allow the surgeon to know which part of the tissue the radiation is coming from, so that they can do accurate node identification or tissue excision with this visual feedback.

## Conclusion

In this paper, we proposed a new hybrid marker which incorporated both circular dots and chessboard vertices to increase the detection rate. The additional green stripe was included to introduce asymmetry and resolve direction ambiguity. The marker was designed such that it fully covered the tethered laparoscopic gamma probe using dense features. The experimental results show that the detection workspace, robustness and pose estimation efficiency and accuracy of the design outperformed previous works. We have therefore shown the feasibility and the potentiality of using the proposed framework to track the ‘SENSEI^®^’ probe. In addition to the design of the new marker, we have also proposed a solution to provide clear visual feedback to indicate the tracer location on the tissue surface.

The work could be further extended to increase the registration accuracy by fusing the vision-based 3D pose estimation with kinematic data of the instrument (robot) controlling the probe. Successive transformations from the probe to the instrument and endoscope coordinate frames will provide a robust initial viewpoint estimate and registration. The framework could also be used to track other types of probes.

## References

[1] C. R. UK. Prostate cancer statistics. https://www.cancerresearchuk.org/health-professional/cancer-statistics/statistics-by-cancer-type/prostate-cancer. Accessed 13 Nov 2019

[2] Trinh Q-D, Sammon J, Sun M, Ravi P, Ghani KR, Bianchi M, Jeong W, Shariat SF, Hansen J, Schmitges J, Jeldres C, Rogers CG, Peabody JO, Montorsi F, Menon M, Karakiewicz PI (2012). Perioperative outcomes of robot-assisted radical prostatectomy compared with open radical prostatectomy: results from the nationwide inpatient sample. Eur Urol.

[3] Strong VE, Humm J, Russo P, Jungbluth A, Wong WD, Daghighian F, Old L, Fong Y, Larson SM (2008). A novel method to localize antibody-targeted cancer deposits intraoperatively using handheld pet beta and gamma probes. Surg Endosc.

[4] Kang X, Azizian M, Wilson E, Wu K, Martin AD, Kane TD, Peters CA, Cleary K, Shekhar R (2014). Stereoscopic augmented reality for laparoscopic surgery. Surg Endosc.

[5] Cheung CL, Wedlake C, Moore J, Pautler SE, Peters TM (2010) Fused video and ultrasound images for minimally invasive partial nephrectomy: a phantom study. In: International conference on medical image computing and computer-assisted intervention. Springer, Berlin, pp 408–415 10.1007/978-3-642-15711-0_51 20879426

[6] Jayarathne UL, McLeod AJ, Peters TM, Chen EC (2013) Robust intraoperative US probe tracking using a monocular endoscopic camera. In: Medical image computing and computer-assisted intervention. Springer, pp 363–370 10.1007/978-3-642-40760-4_46 24505782

[7] Edgcumbe P, Nguan C, Rohling R (2013) Calibration and stereo tracking of a laparoscopic ultrasound transducer for augmented reality in surgery. In: Augmented reality environments for medical imaging and computer-assisted interventions. Springer, pp 258–267

[8] Jayarathne UL, Chen EC, Moore J, Peters TM (2018). Robust, intrinsic tracking of a laparoscopic ultrasound probe for ultrasound-augmented laparoscopy. IEEE Trans Med Imaging.

[9] Pratt P, Jaeger A, Hughes-Hallett A, Mayer E, Vale J, Darzi A, Peters T, Yang GZ (2015). Robust ultrasound probe tracking: initial clinical experiences during robot-assisted partial nephrectomy. Int J Comput Assist Radiol Surg.

[10] Zhang L, Ye M, Chan PL, Yang GZ (2017). Real-time surgical tool tracking and pose estimation using a hybrid cylindrical marker. Int J Comput Assist Radiol Surg.

[11] Bennett S, Lasenby J (2014). ChESS—quick and robust detection of chess-board features. Comput Vis Image Underst.

[12] Neubeck A (2006) Efficient non-maximum suppression. In: 18th International conference on pattern recognition (ICPR’06), vol 3. IEEE

[13] Bouguet J-Y (2001). Pyramidal implementation of the affine lucas kanade feature tracker description of the algorithm. Int Corp.

[14] Collins T, Bartoli A (2014). Infinitesimal plane-based pose estimation. Int J Comput Vis.

[15] Zhang Z (2000). A flexible new technique for camera calibration. IEEE Trans Pattern Anal Mach Intell.

[16] Shah M (2011). Comparing two sets of corresponding six degree of freedom data. Comput Vis Image Underst.

